# Effectiveness of pranayama for mental disorders: a systematic review and meta-analysis of randomized controlled trials

**DOI:** 10.3389/fpsyt.2025.1616996

**Published:** 2025-08-14

**Authors:** Christian Mütze, Dietmar Mitzinger, Heidemarie Haller

**Affiliations:** Center for Integrative Medicine and Planetary Health, University Hospital Essen, University of Duisburg-Essen, Essen, Germany

**Keywords:** pranayama, yogic breathing, yoga, mental disorders, complementary medicine, integrative medicine

## Abstract

**Introduction:**

This review systematically assessed the evidence on the effectiveness and safety of prāṇāyāma, traditional yogic breathing techniques, for patients diagnosed with mental disorders.

**Methods:**

We searched PubMed, PsycINFO, and Central until April 2024. We calculated standardized mean differences (SMDs) with 95% confidence intervals (CIs) from both intention-to-treat (ITT) and per-protocol (PP) data for symptom severity (primary outcome) and health-related quality of life and depression (secondary outcomes) using Hedges’ correction for small samples. For risk of bias (RoB) assessment, we used the Cochrane RoB 2 tool.

**Results:**

We included seven publications on six randomized controlled trials that examined 517 patients with posttraumatic stress disorder (PTSD), depression, and mixed non-psychotic mental disorders and compared prāṇāyāma to passive controls (wait list and attention control) or standard care (psychotherapy, electroconvulsive therapy, and antidepressants). Overall RoB was assessed with some concerns in two studies and as high in four studies. The meta-analyses of the ITT samples (SMD = −0.27, 95% CI = [−0.52, −0.03], I^2^ = 10%) as well as the PP samples (SMD = −0.35, 95% CI = [−0.57, −0.12], I^2^ = 0%) showed that prāṇāyāma significantly reduced post-intervention symptom severity in comparison to passive controls. When compared to standard care, both ITT and PP meta-analyses showed comparable results in reducing symptom severity. For secondary outcomes, only PP analyses on quality of life showed significantly higher post-intervention effects for prāṇāyāma in comparison to passive controls (SMD = 0.59, 95% CI = [0.31, 0.87], I^2^ = 20). No significant effects were found for depression. Sensitivity analyses excluding all studies with a high risk of overall bias revealed significant effects of prāṇāyāma on symptom severity and quality of life, but only in PP samples and in comparison to passive controls. Adverse events were more frequently associated with fast than with slow breathing techniques.

**Discussion:**

This meta-analysis suggests short-term effects of prāṇāyāma when integrated in outpatient and inpatient care of mental disorders. In consideration of the overall high risk of bias and low number of analyzed patients, prāṇāyāma should not be used instead of standard therapies. Further research is needed to explore long-term effects and adequately assess adverse events.

**Protocol registration at Prospero:**

CRD42024550239

## Introduction

The rapid increase in psychological disorders over recent decades has become a critical global challenge, profoundly affecting healthcare systems, societies, and individuals worldwide (1). Psychological disorders, such as depression, anxiety, and posttraumatic stress disorder (PTSD), are among the leading causes of disability on a global scale (2). Lifetime prevalence rates are significant, impacting 28.6% of men and 29.8% of women, with the risk of developing a mental disorder by age 75 reaching 46.4% for men and 53.1% for women (3).

Conventional treatment approaches for psychological disorders, such as cognitive–behavioral therapy (CBT) and pharmacological interventions, are widely recommended as first-line treatments. However, their efficacy often remains modest, with meta-analyses revealing effect sizes of 0.34 for CBT and 0.36 for medication in comparison to treatment-as-usual or placebo (4). Pharmacological treatments, moreover, are frequently associated with side effects such as weight gain, fatigue, and sexual dysfunction (5). As mental health disorders continue to rise, we need more innovative and integrative treatment approaches combining standard therapies with evidence-based practices from traditional Chinese, Indian, or European medicine (6). With nearly half of the population in developed countries and similar or higher proportions in developing nations, the interest in and use of complementary treatments is evident (7), in particular in mental disorders (8), highlighting the need for research to evaluate the effectiveness and safety of complementary and integrative medicine (CIM).

Among CIM approaches, yoga is recognized for its accessibility, low risk of side effects, and potential benefits for stress and mental health (9–11). Similarly, breathwork techniques have demonstrated significant effects on self-reported stress, anxiety, and depression (12). However, no meta-analysis has specifically evaluated prāṇāyāma—the Sanskrit term for breath-based yoga techniques. We therefore aimed to evaluate the effectiveness and safety of prāṇāyāma in improving mental health outcomes in individuals diagnosed with mental disorders according to the *Diagnostic and Statistical Manual of Mental Disorders* (DSM) or the *International Classification of Diseases* (ICD).

## Materials and methods

This review followed the updated Preferred Reporting Items for Systematic Reviews and Meta-Analyses (PRISMA) guidelines (13) and adhered to the Cochrane Collaboration’s recommendations (14). We pre-registered the methodology on PROSPERO (CRD42024550239).

### Eligibility criteria

Studies were eligible if they were randomized controlled trials (RCTs) or clinical controlled trials (CCTs) with or without cross-over design, including adult patients (≥18 years) diagnosed with mental disorders as defined by ICD or DSM criteria or assessed through validated self-report measures. Due to the different diagnostic criteria that apply to children and adolescents, these samples were not considered. Studies were further included if prāṇāyāma techniques were applied as a standalone intervention, excluding studies that combined prāṇāyāma with other yoga components (except for relaxation) or other treatments that were not provided to both of the groups. There were no predefined criteria for intervention duration or frequency to capture a comprehensive range of the existing evidence. Eligible control interventions consisted of inactive and active comparators such as treatment as usual, wait list, attention control interventions, pharmacological treatments, or other non-pharmacological comparators. To be included, studies had to report at least one of the following outcomes: the primary outcome was symptom severity of the primary diagnosis, while secondary outcomes included health-related quality of life, depression, and safety. When multiple instruments were used to assess the same outcome in a study, preference was given to disease-specific over generic instruments, multi-item over single-item measures, and clinician-reported over patient-reported tools. Beyond that, the primary outcomes of the individual studies were favored over secondary ones. Safety was operationalized by the number of adverse events (AEs) or study withdrawals due to AEs. AEs were defined as any unfavorable medical event experienced by a participant, regardless of whether it was caused by the intervention. Events leading to death, life-threatening conditions, hospitalization, or substantial disability were classified as serious AEs (15).

### Literature search

We systematically searched PubMed, PsycINFO, and the Cochrane Central Register of Controlled Trials until April 2024 without language or date restrictions. The search strategy (**Table 1**) included mesh and title/abstract searching for yoga and prāṇāyāma terms combined with those for CCTs and RCTs and was adapted for each of the databases. We also searched gray literature through ResearchRabbit and international trial registries of the National Institutes of Health (NIC) and World Health Organization (WHO). Following the PRISMA guidelines (13), two reviewers (CM and HH) independently screened titles and abstracts of studies and assessed full texts for eligibility. Any disagreements were rechecked with a third reviewer (DM) until consensus was reached.

**Table 1:** Search terms for PubMed.

#1	(yoga[Mesh] AND (breath*[tiab] OR respirat*[tiab] OR diaphragm*[tiab])) OR (breathing exercises[Mesh] AND (yoga[tiab] OR yogic[tiab] OR prana*[tiab])) OR (respiration therapy[Mesh] AND (yoga[tiab] OR yogic[tiab] OR prana*[tiab])) OR prana*[tiab] OR (yoga[tiab] AND (breath*[tiab] OR respirat*[tiab] OR diaphragm*[tiab])) OR (yogic[tiab] AND (breath*[tiab] OR respirat*[tiab] OR diaphragm*[tiab]))
#2	randomized controlled trial [pt] OR controlled clinical trial [pt] OR random* [tiab] OR placebo [tiab] OR sham [tiab] OR trial* [tiab] OR group* [tiab]
#3	#1 AND #2

### Data extraction

Two reviewers (CM and DM) independently extracted data on study characteristics, including trial type, study origin, population characteristics, randomized sample size, mean age ± standard deviation (SD), percentage of female participants, treatment duration, intervention details, assessment points, outcomes included and not included in the meta-analysis, and funding sources. Discrepancies were rechecked by a third reviewer (HH) until consensus was reached. Missing study data were requested from the study authors by email.

### Risk of bias in individual studies

The revised Cochrane risk-of-bias tool (RoB 2) (16) was used to assess the risk of bias in each included study across five domains: 1) bias arising from the randomization process, 2) bias due to deviations from intended interventions, 3) bias due to missing outcome data, 4) bias in the measurement of the outcome, and 5) bias in the selection of the reported result. Two reviewers (CM and DM) independently assessed each domain, and any discrepancies were resolved through discussion with a third reviewer (HH) to reach consensus. Each domain was rated as “low risk of bias”, “some concerns”, or “high risk of bias” according to the RoB 2 guidelines. An overall risk-of-bias judgment was derived based on domain-level ratings. Bias impact was visualized using the RoBvis tool (17).

### Risk of bias across studies

We initially planned to evaluate the evidence of publication bias using Egger’s test. However, since the number of included studies for each meta-analysis was fewer than 10, it was not possible to assess publication bias.

### Qualitative and quantitative synthesis

The qualitative synthesis included a detailed comparison of study characteristics and risk of bias. As safety data were reported insufficiently, we decided against meta-analyzing data and reported AEs qualitatively.

#### Quantitative synthesis of overall effect sizes

A pairwise meta-analysis was performed using Cochrane Review Manager (RevMan, Version 5.4.1). Where studies provided the same type of intervention and category of comparator (passive controls versus standard care), with the same outcome (which can be measured by different instruments) at the same assessment point (post-intervention and latest follow-up), the results were pooled using random-effects models to account for expected heterogeneity across the included studies. This approach assumes that true effect sizes may vary between studies due to clinical or methodological differences and estimates an average effect while incorporating between-study variance into the weighting of individual studies. Effects were displayed as standardized mean differences (SMDs) and 95% confidence intervals (CIs), adjusted by Hedges’ correction for small samples (14). Following Cohen’s benchmarks (18), Hedges’ g values were interpreted as small (0.2–0.5), medium (0.5–0.8), or large (>0.8), with respective readings for negative SMDs. For symptom severity and depression, a negative SMD indicated a greater improvement in prāṇāyāma compared to controls, whereas a positive SMD indicated greater improvements in quality of life. Heterogeneity between effect estimators was assessed using the chi^2^ test and the I^2^ statistic. An I^2^ value greater than 50% was considered indicative of substantial heterogeneity (14, 19).

#### Subgroup analyses

We considered analyzing subgroups of patients who fully adhered to the intervention protocol [per-protocol (PP)] in contrast to all patients who were intended to be treated regardless of whether they completed the outcome assessment or adhered fully to the intervention [intention-to-treat (ITT)]. Initially, we also planned to analyze subgroups of patients with different mental disorders. However, due to the low number of available studies, this was not possible.

#### Sensitivity analyses

We conducted sensitivity analyses based on study quality for studies with a high risk of bias versus low risk/some concerns of overall bias. If at least substantial statistical heterogeneity was present in a respective meta-analysis, we used sensitivity analyses as well to explore heterogeneity in effect estimates according to study quality, study populations, and intervention characteristics.

## Results

### Literature search

**Figure 1:**
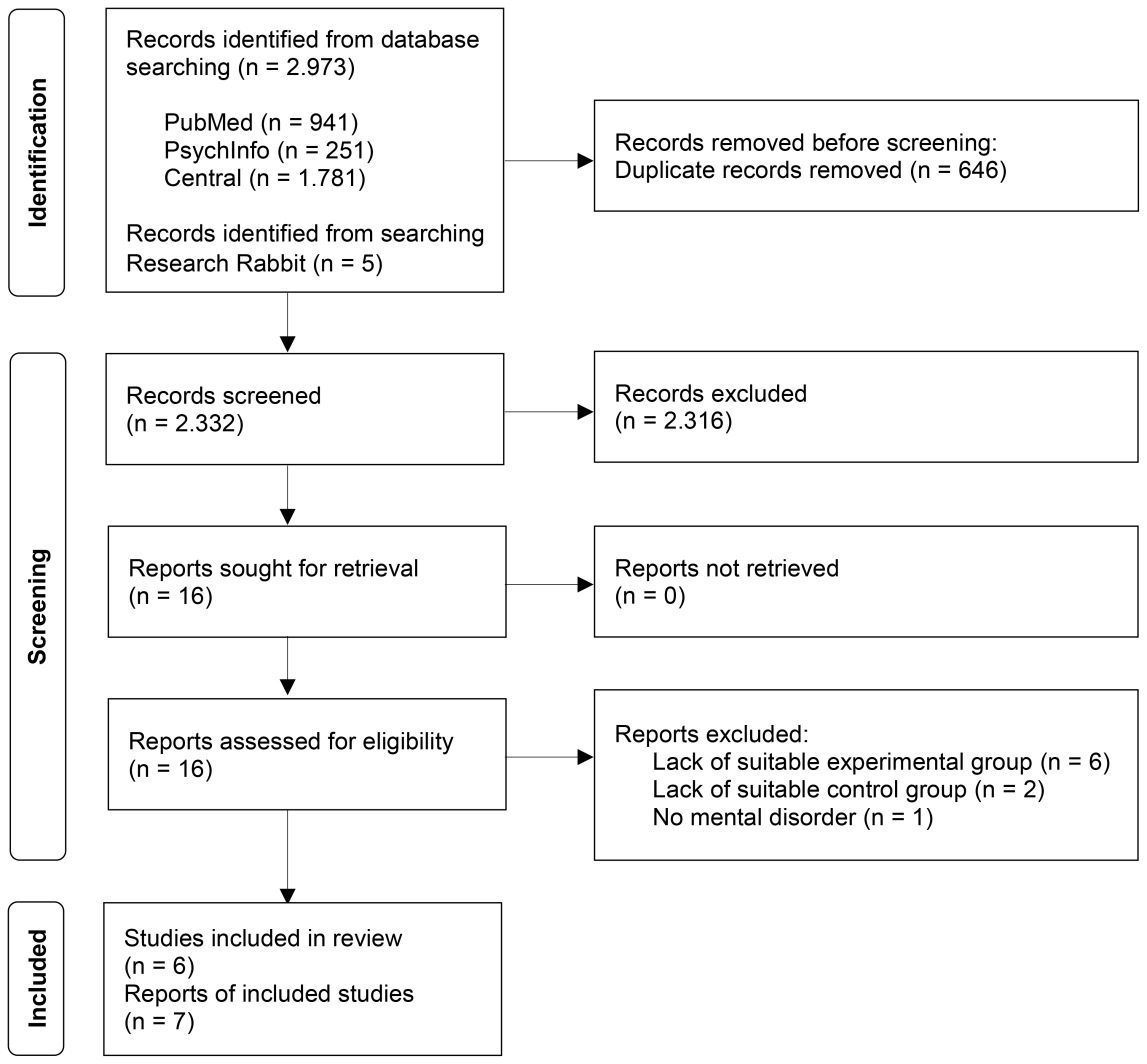
Flowchart of the literature search.

The electronic database search revealed 2,973 articles (**Figure 1**). Five additional articles were retrieved from ResearchRabbit. After removing duplicates and excluding articles by screening titles and abstracts, 16 full-text articles were assessed for eligibility. Nine full texts were excluded for the following reasons: six articles examined prāṇāyāma combined with other yoga elements or interventions (20–25), two articles lacked suitable control groups by including healthy adults or no control condition (26, 27), and one article included participants with burnout, which is not defined as a mental disorder (28). Thus, a final sample of seven articles on six individual datasets, including 517 patients, was included for qualitative and quantitative synthesis (29–35).

### Study characteristics

The characteristics of the included studies are presented in **Table 2**. All studies were RCTs conducted between 2000 and 2023 in the USA (29, 30, 33), India (32, 34, 35), and Germany (31). Patients were civilians (31, 32, 35), veterans (29, 33), and prisoners (34) suffering from various mental disorders, including PTSD (29–31, 33), depression (32), alcohol dependence disorder (35), and mixed non-psychotic mental disorders (34). Sample sizes varied from 21 to 232, with a median of 67 participants and a median of 6% female participants. The average age across studies was 40.2 years, ranging from 28.7 to 56.9 years. The duration and frequency of prāṇāyāma sessions varied by setting and population between 1 and 6 weeks, with an average of 17.5 sessions, while one study did not report duration in weeks but in sessions of 10 (31). Six out of the seven studies implemented Sudarśan Kriyā Yoga (SKY) as a standardized protocol (29, 30, 32–35), although the frequency and types of breathing exercises varied from low to fast/high frequency. The remaining study used a tailored protocol consisting of four specific prāṇāyāma exercises (31): one fast breathing technique of high frequency, two slow-breathing ones, and one breath-holding technique (**Table 3**). Control conditions included passive ones such as wait list (33, 35) and attention control (34) as well as standard treatments such as cognitive processing therapy (29, 30), electroconvulsive therapy (32), and antidepressant drugs (32). In the latter study, the data of the two control groups were combined to avoid biasing the final sample size. Similarly, one study (31) provided two measurements for the same endpoint (physical and psychological quality of life) that were pooled to provide a single value.

**Table 2:** Study characteristics.

Reference	Trial type	Origin	Population	Sample size	Female	Mean age ± SD	Treatment duration	Assessment points	Treatment group	Control group	Outcomes included in meta-analysis	Outcomes not included in meta-analysis	Funding
Bayley (2022) (29) and Schulz-Heik (2022) (30)	RCT	USA	Veteran outpatients with PTSD + comorbid AD, AUD, and SUD	85	12%	56.9 **±** 12.8	5 weeks	PI: 6 weeks FU: 12 months	Sudarśan Kriyā Yoga	Cognitive processing therapy	−Symptom severity (PCL-5)^P#^ −Quality of life (VQ-P)^P^ −Depression (BDI-II)^P^ −Safety (AEs)^P^	−PTSD severity (CAPS-5)^C‡^ −Quality of life (VQ-O)^P^	Government
Haller (2023) (31)	RCT	Germany	Civilian outpatients with PTSD	74	84%	44.2 ± 13.0	10 sessions	PI: after 10 sessions	Prāṇāyāma + cognitive behavioral therapy	WL + cognitive behavioral therapy	−Symptom severity (PCL-5)^P#^ −Quality of Life (SF-12)^P^ −Depression (BDI-II)^P^ −Safety (AEs)^P/C^	-None	University
Janakiramaiah (2000) (32)	RCT	India	Civilian inpatients with depression	45	44%	38.7 ± 7.4	4 weeks	PI: 4 weeks	Sudarśan Kriyā Yoga	−Electroconvulsive therapy −Imipramine	−Symptom severity (HDRS)^C#^ −Depression (HDRS)^C#^ −Safety (AEs)^C^	Depression (BDI)^P^	N.r.
Seppälä (2014) (33)	RCT	USA	Veteran outpatients with PTSD	21	0%	28.7 ± 4	1 week	PI: 1 week FU: 48 weeks	Sudarśan Kriyā Yoga	WL	−Symptom severity (PCL-M)^P#^ −Depression (MASQ subscales GDD+AD)^P^ −Safety (AEs)^C^	None	Trust
Sureka (2014) (34)	RCT	India	Prisoners with mixed mental disorders (OCD, GAD, MDD, SD, AJD, and HID)	230	0%	36.1 ± 11.4	6 weeks	PI: 6 weeks	Sudarśan Kriyā Yoga	Attention control (sitting with attention to the breath)	−Symptom severity (GAFP)^C^ −Quality of life (PGWB)^P^ −Safety (AEs)^C^	None	N.r.
Vedamurthachar (2006) (35)	RCT	India	Civilian inpatients with ADS	60	0%	36.7 ± 7.7	2 weeks	PI: 3 weeks	Sudarśan Kriyā Yoga	WL	−Depression (BDI)^P#^ −Safety (AEs)^C^	None	Hospital

AA, anxious arousal; AD, anxiety disorder; AJD, adjustment disorder; AEs, adverse events; ADS, alcohol dependence syndrome; AUD, alcohol use disorder; BDI/II, Beck Depression Inventory/2nd Revision; CAPS-5, Clinician-administered PTSD Scale for DSM-5; FU, latest follow-up; GAD, generalized anxiety disorder; GAF, Global Assessment of Functioning Scale; HDRS, Hamilton Depression Rating Scale; HID, Habit and Impulse Disorder; MDD, major depressive disorder; MASQ-AD/GDD, Mood and Anxiety Symptoms Questionnaire, Anhedonic Depression Subscale/General Distress-Depressive Subscale; N.r., not reported; OCD, obsessive compulsive disorder; PCL-5/M, Posttraumatic Stress Disorder Checklist for DSM-5 Civilian Version/Military Version; PGWB, Psychological General Well Being Schedule; PI, post-intervention; PTSD, posttraumatic stress disorder; RCT, randomized controlled trial; SD, somatoform disorder; SF-12, Short Form 12 Health Survey; SUD, substance use disorder; VQ-P, Valuing Questionnaire Progress; VQ-O, Valuing Questionnaire Obstruction; WL, wait list.

Special characters: ^P^, patient-reported outcome; ^C^, clinician-administered outcome; ^#^, Primary outcome(s) of the study; ^‡^, Data not published and trial authors did not respond to email request.

**Table 3:** Detailed characteristics of prāṇāyāma interventions.

Reference	Prāṇāyāma techniques used	Session characteristics	Setting	Instructor background
Bayley (2022) (29) and Schulz-Heik (2022) (30)	−Nāḍī śodhana (slow) −Śītalī (slow) −Ujjāyī (slow) −Bhastrikā (fast) −Sudarśan kriyā (slow and fast)	Initial 5-day group workshop (3 hours per day) + 10 sessions of 60 min, twice weekly over 6 weeks	Outpatient, group setting	Certified instructors by the Project Welcome Home Troops
Haller (2023) (31)	−Nāḍī śodhana (slow) −Ujjāyī (slow) −Kapālabhātī (fast) −Kumbhaka (retention)	10 sessions of 5–10 min over consecutive therapy units	Outpatient, one-to-one setting	Psychotherapists with additional training in yoga therapy
Janakiramaiah (2000) (32)	−Ujjāyī (slow) −Bhastrikā (fast) −Sudarśan kriyā (slow and fast)	4–6×/week over 4 weeks of 30 min	Inpatient, group setting	Yoga teacher trained by the Art of Living Foundation
Seppälä (2014) (33)	−Ujjāyī (slow) −Bhastrikā (fast) −Sudarśan kriyā (slow and fast)	3 ×1-hour group sessions daily over 7 days	Outpatient, group setting	Certified instructors by the Project Welcome Home Troops
Sureka (2014) (34)	Nāḍī śodhana (slow) −Ujjāyī (slow) −Bhastrikā (fast) −Sudarśan kriyā (slow and fast)	5 sessions per week of 30 min over 6 weeks	Prison, group setting	Certified yoga teachers by the Art of Living Foundation
Vedamurthachar (2006) (35)	−Ujjāyī (slow) −Bhastrikā (fast) −Sudarśan kriyā (slow and fast)	7 sessions of 45–60 min (every other day) over 2 weeks	Inpatient, group setting	Therapists trained by the Art of Living Foundation

Explanation of Sanskrit names: Bhastrikā, bellows breathing; Kapālabhātī, passive inspiration with forceful expiration; Kumbhaka, holding of the breath until the breathing reflex prevailed over volition; Nāḍī śodhana, alternate nostril breathing; Śītalī, straw breathing; Ujjāyī, deep breathing with trachea contraction; Sudarśan kriyā, rhythmic cyclic breathing.

Regarding data analysis, two studies presented findings for both PP and ITT populations: one applying the last observation carried forward method (29) and the other using fully conditional specification iterations (31). Four studies reported values that could be interpreted as ITT ones due to no (32, 35) or minimal dropout rates of n = 1 per group (33, 34), while one study (30) conducted only exploratory PP analysis.

### Risk of bias of individual studies

RoB is summarized in **Table 4**. Overall, RoB was high in four studies (29, 30, 32, 33, 35) and assessed with some concerns in two studies (31, 34). Regarding bias from the randomization process, only one study (31) provided a clear description of the allocation procedure. In contrast, one study (29, 30) excluded participants directly after randomization without explanation, potentially introducing bias. For the remaining studies (32–35), information was insufficient to adequately assess the entire randomization process.

**Table 4:** Risk of bias of individual studies.

	D1	D2	D3	D4	D5	Overall
Bayley (2022) (29) and Schulz-Heik (2022) (30)	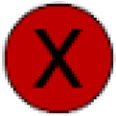	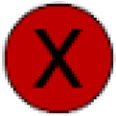	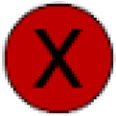	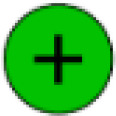	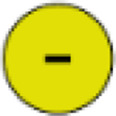	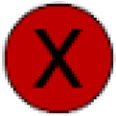
Haller (2023) (31)	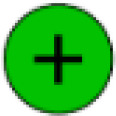	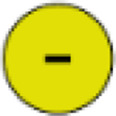	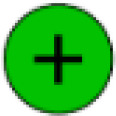	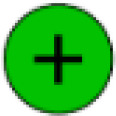	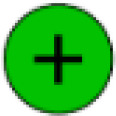	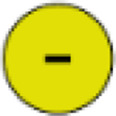
Janakiramaiah (2000) (32)	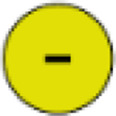	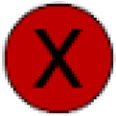	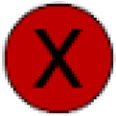	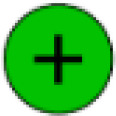	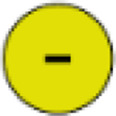	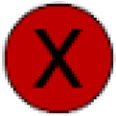
Seppälä (2014) (33)	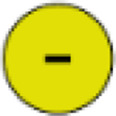	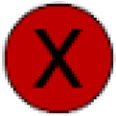	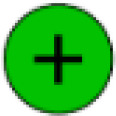	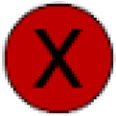	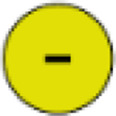	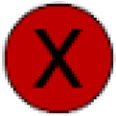
Sureka (2014) (34)	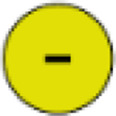	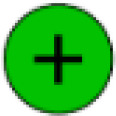	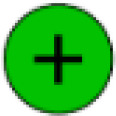	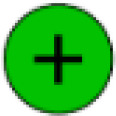	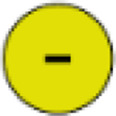	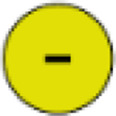
Vedamurthachar (2006) (35)	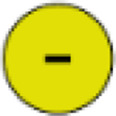	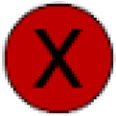	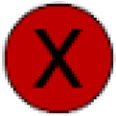	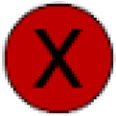	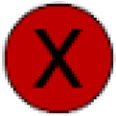	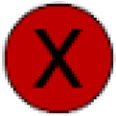

Domains: D1, bias due to the randomization process; D2, deviation from intended intervention; D3, missing outcome data; D4, measurement of outcomes; D5, selection of the reported result.Judgement: X, High; -, Some concerns; +, Low.

Bias due to deviations from intended interventions was assessed as follows: due to the nature of the prāṇāyāma intervention, participants and providers were aware of assignments in all studies. However, one study (34) ensured scientific rigor by including a suitable attention control group, while one study (31) considered statistical adjustments for patient expectation, raising only some concerns. The other four studies were rated as high risk, as the authors deviated from the protocol by excluding participants after randomization with an incomprehensible explanation (29, 30), did not provide sufficient information (32), or possibly introduced nocebo effects by not offering any control intervention other than wait list (35).

Bias due to missing outcome data was rated low in three studies because of minimal dropout rates (33, 34) or ITT analysis with robust imputation methods of missing data (31). The remaining three studies were rated as high risk, as they did not provide sufficient information (32, 35) or excluded participants without justification (29, 30).

Measurement bias was generally low, as all studies used validated instruments with consistent methods and time points. However, in two studies (33, 35), the absence of active control conditions may have influenced outcome assessments, indicating a high risk of bias.

Finally, selective outcome reporting was low in only one study (31) that fully adhered to a registered protocol. Four studies raised some concerns, as they differed slightly from the published protocol without adequate explanation (29, 30) or did not report pre-registered protocols but reported comprehensible outcome selection, which do not seem to be biased (32–34). One study, however, reported using the Severity of Alcohol Dependence Questionnaire at baseline and follow-up, but only presented baseline values (35).

### Quantitative synthesis

#### Effects on symptom severity

**Figure 2:**
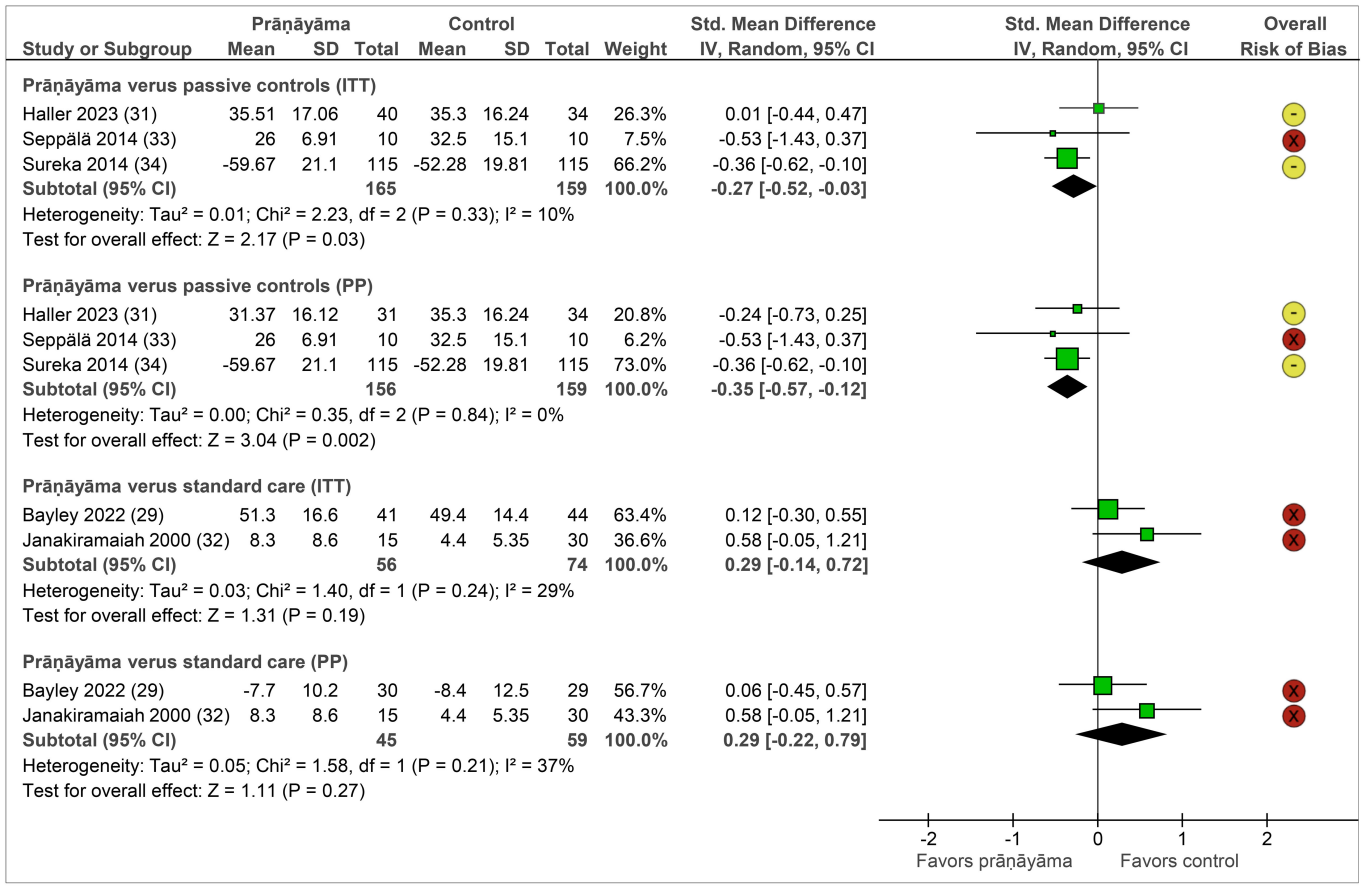
Forest plot of symptom severity. Legend: I^2^, heterogeneity; ITT, intention-to-treat population; CI, confidence interval; IV, inverse variance; PP, per-protocol population; SD, standard deviation. Note: Passive controls include wait list (31, 33) and attention control (34) groups; standard care includes cognitive processing therapy (29), electroconvulsive therapy (32), and imipramine (32).

Symptom severity was measured by clinician-administered and patient-reported instruments in patients with PTSD, depression, and mixed mental disorders. Compared to passive controls post-intervention, prāṇāyāma resulted in significantly greater symptom reductions of small effect sizes in ITT samples (SMD = −0.27, 95% CI = [−0.52, −0.03], I^2^ = 10%, N = 324) as well as in PP ones (SMD = −0.35, 95% CI = [−0.57, −0.12], I^2^ = 0%, N = 315) (31, 33, 34). When compared to standard care, meta-analyses in both ITT and PP samples showed non-significantly different results to prāṇāyāma (**Figure 2**).

Follow-up data on PTSD severity were reported by two studies with different control conditions up to 12 months. One study (33) comparing prāṇāyāma to wait list found a significant group–time interaction, and the other one (29) reported non-inferiority of prāṇāyāma against cognitive processing therapy.

#### Effects on secondary outcomes

**Figure 3:**
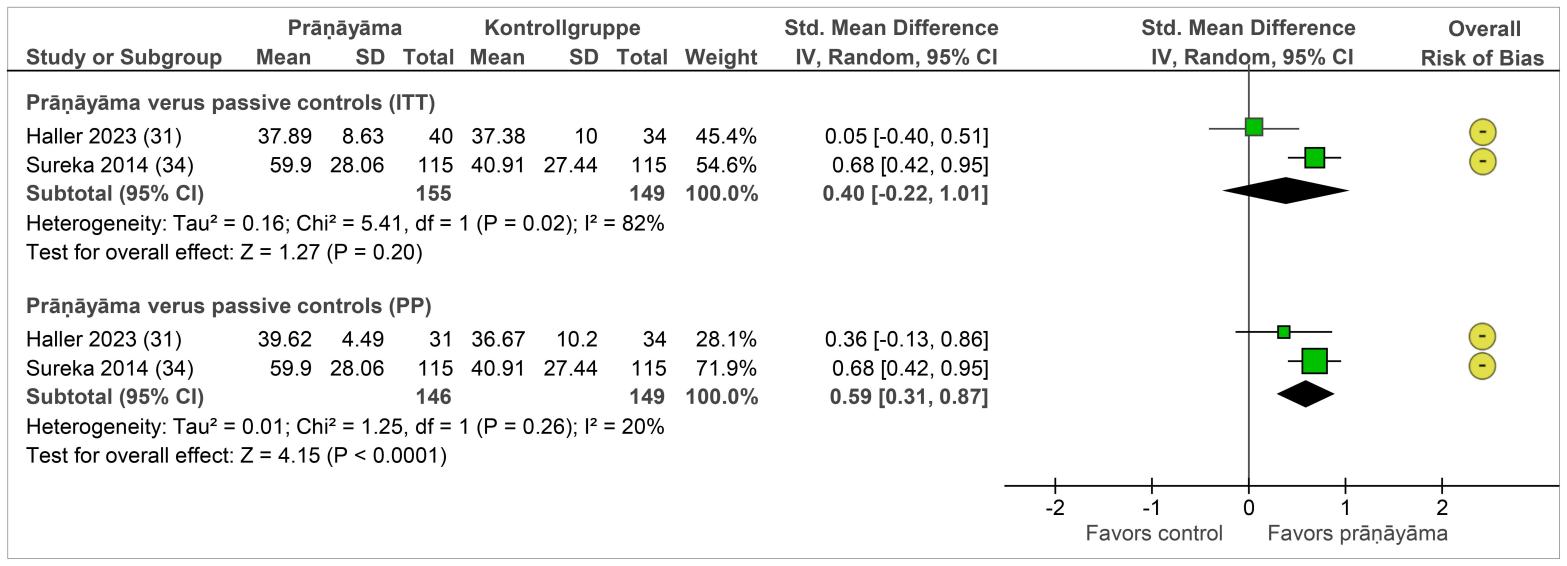
Forest plot of quality of life. Legend: I^2^, heterogeneity; ITT, intention-to-treat population; CI, confidence interval; IV, inverse variance; PP, per-protocol population; SD, standard deviation. Passive controls include wait list (31) and attention control (34) groups.

Quality of life was assessed using patient-reported instruments in samples with PTSD and mixed mental disorders. Meta-analyses could be performed only for comparisons of prāṇāyāma against passive controls and are shown in **Figure 3**. Directly after the end of the intervention, prāṇāyāma did not show significantly different effects in the ITT samples but did so in the PP samples (SMD = 0.59, 95% CI = [0.31, 0.87], I^2^ = 20%, N = 295) (31, 34). One further study reported quality of life data post-intervention against cognitive processing therapy and did not find statistically significant differences (30). For this outcome, follow-up data were not available.

**Figure 4:**
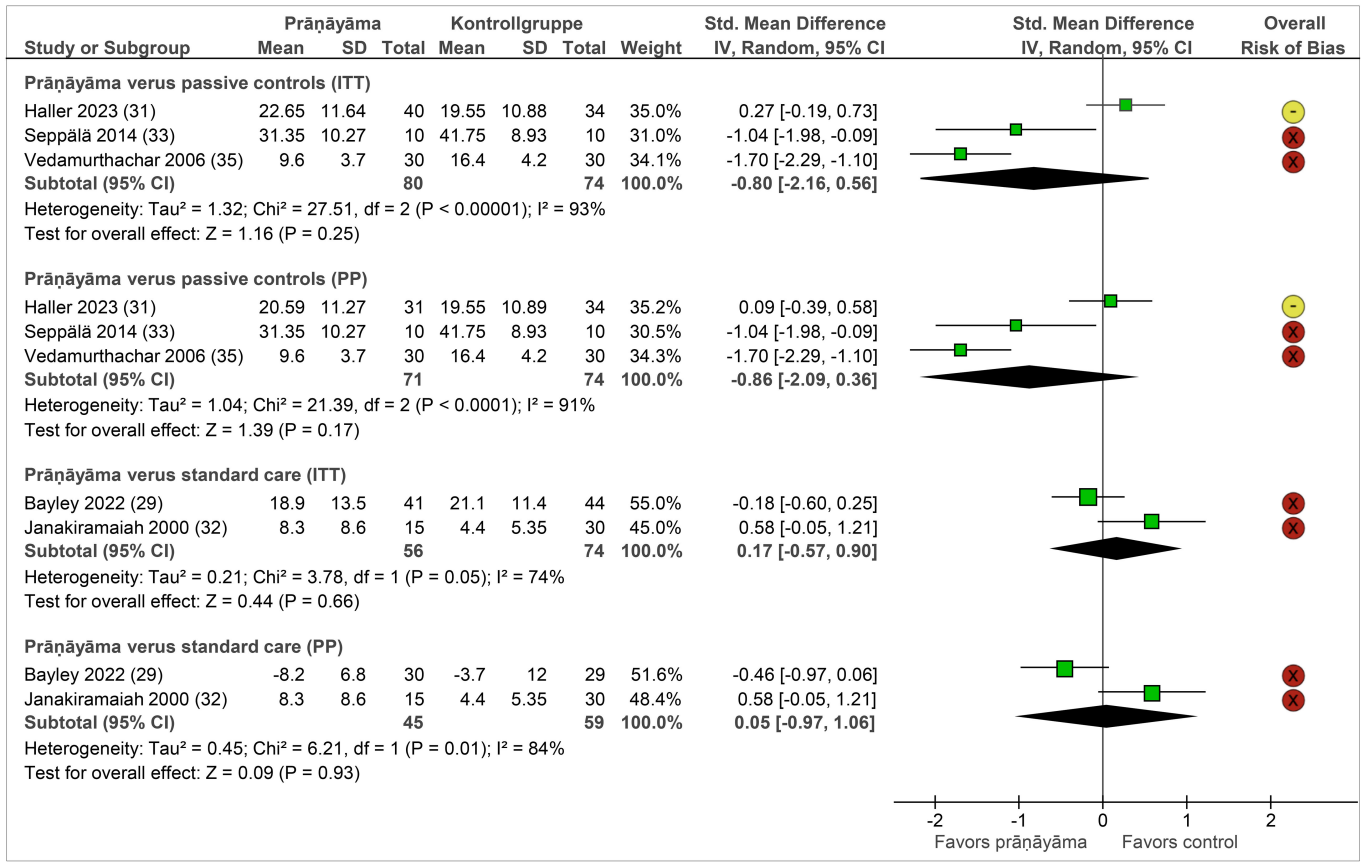
Forest plot of depression. Legend: I^2^, heterogeneity; ITT, intention-to-treat population; CI, confidence interval; IV, inverse variance; PP, per-protocol population; SD, standard deviation. Passive controls include wait list (31, 33, 35), and standard care includes cognitive processing therapy (29), electroconvulsive therapy (32), and imipramine (32).

Depression was assessed using clinician-administered and patient-reported instruments in participants with PTSD, depression, and alcohol dependence disorder. The pooled effects on depression are shown in **Figure 4**. Compared with passive controls as well as standard care, prāṇāyāma did not show any significant differences. Follow-up data were reported by two studies showing no significant group differences up to 12 months.

Safety data were reported insufficiently. Thus, we decided against meta-analyzing data and reported AEs qualitatively. While one study (31) assessed AEs and drop-outs by AEs systematically, the others did not. In this study, one patient of the wait list reported fatigue, while 15 patients of the prāṇāyāma group reported minor AEs such as anxiety, breathlessness, cough, dizziness, feeling of constriction, flashback, headache, and neck pain, which had high probability of being associated with prāṇāyāma, in particular with kapālabhātī, a fast/high-frequency breathing technique. Although these AEs were classified as minor and not recurrent, they led to drop-outs and significantly worse effects on selected study outcomes when compared to control patients. Of the 15 prāṇāyāma patients with AEs, three AEs were serious ones and led to hospitalization in two patients (orofacial cleft surgery and stent implantation) and death in one patient (congenital heart defect), but were probably not associated with prāṇāyāma (31). Another study reported no AEs within the prāṇāyāma group and two within the group receiving cognitive processing therapy, of which one was considered serious (hospitalization for suicidal ideation) (29). The remaining studies (32–35) reported no adverse events in either group.

### Sensitivity analyses

By excluding studies with a high risk of overall bias, only comparisons against passive controls remain in the analyses. For symptom severity, pooling effects of ITT samples no longer revealed significant group differences, and statistical heterogeneity increased (SMD = −0.22, 95% CI = [−0.57, 0.13], I^2^ = 48%, N = 304) (31, 34). By pooling the effects of PP samples, differences remain significant (SMD = −0.33, 95% CI = [−0.56, 0.10], I^2^ = 0%, N = 295) (31, 34). For quality of life, all main analyses were based on studies without a high risk of bias. Depression analyses are all based on studies with a high risk of bias, with one exception (31). Comparing the results of this individual study with the pooled effects, all revealed no significant difference between prāṇāyāma and passive controls on depression.

Substantial statistical heterogeneity was observed in meta-analyses of quality of life and depression. As most analyses included only two studies, reducing heterogeneity by excluding studies was only possible for the comparison of prāṇāyāma versus passive controls on depression. Here, statistical heterogeneity could only be reduced by excluding the study with the best quality and the highest number of patients, resulting in significant group differences within the ITT and PP samples. However, as these results are likely subject to substantial bias, the data were not shown.

## Discussion

### Summary of evidence

The systematic search revealed seven RCTs on six samples investigating the effectiveness of prāṇāyāma in patients diagnosed with mental disorders. Based on analyses of studies with overall some concerns of risk of bias, the evidence suggests that prāṇāyāma may have small-to-medium short-term effects on symptom severity and quality of life when compared to passive controls. However, sensitivity analysis showed that when high-risk trials are excluded, the effects remain significant only for PP data, not ITT ones. Moreover, we did not find any effects of prāṇāyāma on depression. Comparable effects of prāṇāyāma against standard psycho- or pharmacotherapy should be interpreted with caution, as they were based on single or pooled high-risk studies. The same is true for long-term effects. Safety was reported insufficiently. Based on the reported data, prāṇāyāma, in particular fast breathing, was associated with adverse events.

### Discussion of results

These findings align with a comprehensive systematic review and meta-analysis of 26 RCTs on breathwork and mental health in mixed populations of healthy individuals and somatically and mentally ill patients who revealed small-to-medium effects of modern and traditional forms of breathwork on self-reported stress, anxiety, and depression compared to non-breathwork active and passive controls (12). Likewise, our meta-analyses did not find any effects on depression. However, due to substantial heterogeneity in all of the analyses, this conclusion should be considered preliminary. The review further reported comparable effect sizes for different breathing techniques, in particular slow and fast breathing exercises, but only slow breathing resulted in significant pooled effects and was therefore recommended to patients. In our analyses, we were not able to differentiate between slow and fast prāṇāyāma techniques because of the overall low number of studies and the combination of low and fast techniques in all of the included studies. However, we detected higher AEs of fast breathing techniques, especially kapālabhātī, which strengthened the prior recommendation. Another scoping review on breathwork for clinically diagnosed anxiety disorders also found significant symptom improvements, particularly with slow diaphragmatic breathing, while the results for fast hyperventilation techniques were contradictory (36). This can be explained by evidence showing that slow breathing enhances parasympathetic activity, improves vagal tone, and regulates emotional responses by modulating brain regions such as the prefrontal cortex and amygdala (37). Furthermore, slow breathing has been associated with increased alpha power in the electroencephalogram (EEG), reflecting states of relaxation and focused attention (38). These physiological mechanisms likely underpin the reductions in anxiety and stress observed following slow breathing interventions and suggest that prāṇāyāma protocols emphasizing slow and controlled breathing techniques could improve therapeutic outcomes while minimizing adverse events associated with fast practices such as kapālabhātī. Nonetheless, fast breathing techniques have shown interesting effects as well. Although they did not lower cortisol levels initially but increased them, fast breathing showed a better long-term stabilization of cortisol levels and was discussed to act as a training impulse to the autonomic nervous system, similarly to physical exercises (39). By communicating possible AEs of fast breathing techniques to patients and adequately accompanying them, patients may equally or even better benefit from fast breathing techniques.

### Limitations of the review

The first limitation is the small number of included studies with limited follow-up data. Conclusions, especially from meta-analyses that include only two RCTs, should therefore be considered preliminary. Substantial heterogeneity in some of the analyses suggests that additional studies are urgently needed to make precise and robust conclusions about the effectiveness of prāṇāyāma, especially as a single intervention against standard care. Moreover, the absence of long-term data in all but two studies (29, 33) limits the conclusions regarding the sustainability of the effects. In addition, given the limited number of studies, we were unable to assess the risk of publication bias, which cannot be excluded and should be considered in the interpretation of the findings.

A second limitation includes the risk of bias profile of the included studies. Although it was comparable with the profile of studies on conventional breathing interventions (12), only 33.3% of the domains could be assessed as low risk, and 27.8% raised some concerns, potentially impacting the internal validity of the studies as well as the generalization of the pooled results.

The third limitation refers to possible placebo effects that cannot be excluded because therapists cannot be blinded to treatment allocation, and analyses contained patient-reported outcomes, which may have been influenced by subjective perceptions and expectations. Only one study (31) controlled for patient expectations in statistical analyses. In contrast, none of the studies addressed other non-specific treatments and therapist effects, such as the therapeutic alliance, which can be controlled, for example, by using validated questionnaires (40).

Additional limitations include the insufficient reporting of adverse events and more favorable effects in small, high-risk studies from India (41), which may bias conclusions regarding the risk–benefit ratio. Moreover, the predominance of male participants (94%) limits the generalizability of findings to female populations.

### Implications for further research

Future studies should investigate both the efficacy and specific mechanisms of prāṇāyāma in treating various psychological disorders in detail. Systematic reporting of AEs is essential to improve comparability and ensure a comprehensive understanding of prāṇāyāma’s safety in the treatment of mental disorders. Future studies should therefore include reporting of AEs according to the Consolidated Standards of Reporting Trials (CONSORT) extension for harms to ensure the standardized reporting of adverse events (42). The sustainability of treatment outcomes should also be evaluated by examining long-term effects. Recommendations further include the need for larger sample sizes, rigorous study designs, and the reporting of methodologies in accordance with CONSORT (43) to minimize the risk of bias. In addition, the Checklist Standardising the Reporting of Interventions For Yoga (CLARIFY) checklist (44) provides researchers with a minimum reporting template for yoga studies. If blinding of therapists is not feasible, attention effects could be controlled by asking patients about their perception of the therapeutic alliance using validated questionnaires. Moreover, further research should include diverse demographic groups. In this review, women were severely underrepresented, with a median of only 6%. Given the higher prevalence of mental health disorders in women (27.2%) than in men (18.1%) (45), this proportion should be accounted for in future studies. Moreover, future research should include more diverse populations beyond the currently studied groups (e.g., Indian clinical samples and U.S. veterans) to improve generalizability across cultural and demographic contexts. Future studies should also aim to clarify specific mechanisms of action by examining factors such as type of prāṇāyāma (e.g., slow versus fast), session duration, intensity, and frequency. Additionally, future trials may directly compare prāṇāyāma with established treatment approaches such as cognitive behavioral therapy or pharmacotherapy to determine comparative effectiveness. Together, these implications will contribute to drawing better conclusions about the efficacy, effectiveness, and safety of prāṇāyāma for patients with mental disorders.

### Implications for clinical practice

prāṇāyāma, in particular slow breathing techniques, may decrease symptom severity and improve quality of life of non-psychotic mental disorders when integrated in out- and inpatient care. To date there is no robust evidence that prāṇāyāma programs has comparable effects to standard treatments such as Cognitive Behavioral Therapy or Antidepressants and should therefore not be used instead of these therapies. Due to the considerable variability in the duration and frequency of prāṇāyāma interventions across studies, no evidence-based recommendation regarding an optimal length or intensity of prāṇāyāma can be made yet. Therapists should also be aware of possible side effects of fast breathing techniques, especially in patients with PTSD or anxiety disorders who might perceive these techniques as additional stressors or even trigger. Nevertheless, they might be useful to increase stress tolerance equally to the use of breath-holding tasks (46).

## References

[1] GBD 2019 Mental Disorders Collaborators. Global, regional, and national burden of 12 mental disorders in 204 countries and territories, 1990-2019: a systematic analysis for the Global Burden of Disease Study 2019. Lancet Psychiatry. (2022) 9:137–50. doi: 10.1016/S2215-0366(21)00395-3, PMID: 35026139 PMC8776563

[2] World Health Organization. Depression and other common mental disorders: global health estimates. Geneva: World Health Organization (2017).

[3] McGrath JJ Al-Hamzawi A Alonso J Altwaijri Y Andrade LH Bromet EJ. Age of onset and cumulative risk of mental disorders: a cross-national analysis of population surveys from 29 countries. Lancet Psychiatry. (2023) 10:668–81. doi: 10.1016/S2215-0366(23)00193-1, PMID: 37531964 PMC10529120

[4] Leichsenring F Steinert C Rabung S Ioannidis JPA. The efficacy of psychotherapies and pharmacotherapies for mental disorders in adults: an umbrella review and meta-analytic evaluation of recent meta-analyses. World Psychiatry. (2022) 21:133–45. doi: 10.1002/wps.20941, PMID: 35015359 PMC8751557

[5] Uher R Farmer A Henigsberg N Rietschel M Mors O Maier W. Adverse reactions to antidepressants. Br J Psychiatry. (2009) 195:202–10. doi: 10.1192/bjp.bp.108.061960, PMID: 19721108

[6] Sarris J Gerbarg PL Brown RP Muskin PR Tasman A Riba MB. Integrative and complementary medicine in psychiatry. In: Tasman A Riba MB Alarcón RD Alfonso CA Kanba S Lecic-Tosevski D, editors. Tasman’s psychiatry. Springer International Publishing, Cham (2024). p. 4537–94.

[7] Seetharaman M Krishnan G Schneider RH. The future of medicine: frontiers in integrative health and medicine. Medicina (Kaunas). (2021) 57(12):1079. doi: 10.3390/medicina57121303, PMID: 34946248 PMC8707659

[8] Melzer J Deter H-C Uehleke B. CAM in psychiatry. Evidence-Based Complementary Altern Med. (2013) 2013:293248. doi: 10.1155/2013/293248, PMID: 23983776 PMC3745881

[9] Cramer H Anheyer D Lauche R Dobos G. A systematic review of yoga for major depressive disorder. J Affect Disord. (2017) 213:70–7. doi: 10.1016/j.jad.2017.02.006, PMID: 28192737

[10] Cramer H Anheyer D Saha FJ Dobos G. Yoga for posttraumatic stress disorder - a systematic review and meta-analysis. BMC Psychiatry. (2018) 18:72. doi: 10.1186/s12888-018-1650-x, PMID: 29566652 PMC5863799

[11] Cramer H Lauche R Anheyer D Pilkington K de Manincor M Dobos G. Yoga for anxiety: A systematic review and meta-analysis of randomized controlled trials. Depress Anxiety. (2018) 35:830–43. doi: 10.1002/da.22762, PMID: 29697885

[12] Fincham GW Strauss C Montero-Marin J Cavanagh K. Effect of breathwork on stress and mental health: A meta-analysis of randomised-controlled trials. Sci Rep. (2023) 13:432. doi: 10.1038/s41598-022-27247-y, PMID: 36624160 PMC9828383

[13] Page MJ McKenzie JE Bossuyt PM Boutron I Hoffmann TC Mulrow CD. The PRISMA 2020 statement: an updated guideline for reporting systematic reviews. Br Med J. (2021) 372:n71. doi: 10.1136/bmj.n71, PMID: 33782057 PMC8005924

[14] Higgins JPT Thomas J Chandler J Cumpston M Li T Page MJ. Cochrane handbook for systematic reviews of interventions. 2nd. Chichester (UK): Wiley (2019).

[15] European Medicines Agency. ICH harmonised tripartite guideline E6 (R1). In: Step 5. Note for guidance on good clinical practice (CPMP/ICH/135/95). European Medicines Agency, London (2002).

[16] Sterne JAC Savović J Page MJ Elbers RG Blencowe NS Boutron I. RoB 2: A revised tool for assessing risk of bias in randomised trials. Br Med J. (2019) 366:l4898. doi: 10.1136/bmj.l4898, PMID: 31462531

[17] McGuinness Luke A Higgins JPT. RoBvis: Visualization tool for risk of bias assessments (2024). Available online at: https://www.riskofbias.info/welcome/robvis-visualization-tool (Accessed March 28, 2025).

[18] Ellis PD. The essential guide to effect sizes: statistical power, meta-analysis, and the interpretation of research results. Cambridge: Cambridge University Press (2012).

[19] Higgins JP Thompson SG. Quantifying heterogeneity in a meta-analysis. Stat Med. (2002) 21:1539–58. doi: 10.1002/sim.1186, PMID: 12111919

[20] Carter J Gerbarg P Brown R Ware R Ambrosio C Anand L. Multi-component yoga breath program for Vietnam veteran post traumatic stress disorder: Randomized controlled trial. J Traumatic Stress Disord Treat. (2013) 2:1–10. doi: 10.4172/2324-8947.1000108

[21] Descilo T Vedamurtachar A Gerbarg PL Nagaraja D Gangadhar BN Damodaran B. Effects of a yoga breath intervention alone and in combination with an exposure therapy for post-traumatic stress disorder and depression in survivors of the 2004 South-East Asia tsunami. Acta Psychiatr Scand. (2010) 121:289–300. doi: 10.1111/j.1600-0447.2009.01466.x, PMID: 19694633

[22] Franzblau SH Echevarria S Smith M Van Cantfort TE. A preliminary investigation of the effects of giving testimony and learning yogic breathing techniques on battered women’s feelings of depression. J Interpers Violence. (2008) 23:1800–8. doi: 10.1177/0886260508314329, PMID: 18319369

[23] Katzman MA Vermani M Gerbarg PL Brown RP Iorio C Davis M. A multicomponent yoga-based, breath intervention program as an adjunctive treatment in patients suffering from generalized anxiety disorder with or without comorbidities. Int J Yoga. (2012) 5:57–65. doi: 10.4103/0973-6131.91716, PMID: 22346068 PMC3276935

[24] Ravindran AV McKay MS da Silva TD Tindall C Garfinkel T Paric A. Breathing-focused yoga as augmentation for unipolar and bipolar depression: A randomized controlled trial: Le yoga axé sur la respiration comme traitement d’appoint pour la dépression unipolaire et bipolaire: Un essai randomisé contrôlé. Can J Psychiatry. (2021) 66:159–69. doi: 10.1177/0706743720940535, PMID: 32677851 PMC7918867

[25] Sharma A Barrett MS Cucchiara AJ Gooneratne NS Thase ME. A breathing-based meditation intervention for patients with major depressive disorder following inadequate response to antidepressants: A randomized pilot study. J Clin Psychiatry. (2017) 78:e59–63. doi: 10.4088/JCP.16m10819, PMID: 27898207 PMC5272872

[26] Shetty KT Subbakrishna D Meti B Raju T. Therapeutic efficacy of sudarshan kriya yoga (SKK) in dysthymic disorder. Nimhans J. (1998) 16(1):21–8.

[27] Bhargav H Nagendra HR Gangadhar BN Nagarathna R. Frontal hemodynamic responses to high frequency yoga breathing in schizophrenia: a functional near-infrared spectroscopy study. Front Psychiatry. (2014) 5:29. 24715879 10.3389/fpsyt.2014.00029 PMC3970016

[28] Bhardwaj P Pathania M Bahurupi Y Kanchibhotla D Harsora P Rathaur VK. Efficacy of mHealth aided 12-week meditation and breath intervention on change in burnout and professional quality of life among health care providers of a tertiary care hospital in north India: a randomized waitlist-controlled trial. Front Public Health. (2023) 11:1258330. doi: 10.3389/fpubh.2023.1258330, PMID: 38026380 PMC10646346

[29] Bayley PJ Schulz-Heik RJ Tang JS Mathersul DC Avery T Wong M. Randomised clinical non-inferiority trial of breathing-based meditation and cognitive processing therapy for symptoms of post-traumatic stress disorder in military veterans. Br Med J Open. (2022) 12:e056609. doi: 10.1136/bmjopen-2021-056609, PMID: 36008059 PMC9422818

[30] Schulz-Heik RJ Lazzeroni LC Hernandez B Avery TJ Mathersul DC Tang JS. Valued living among veterans in breath-based meditation treatment or cognitive processing therapy for posttraumatic stress disorder: Exploratory outcome of a randomized controlled trial. Global Adv Integr Med Health. (2022) 11:2164957x221108376. doi: 10.1177/2164957X221108376, PMID: 35770246 PMC9234823

[31] Haller H Mitzinger D Cramer H. The integration of yoga breathing techniques in cognitive behavioral therapy for post-traumatic stress disorder: A pragmatic randomized controlled trial. Front Psychiatry. (2023) 14:1101046. doi: 10.3389/fpsyt.2023.1101046, PMID: 37139325 PMC10150115

[32] Janakiramaiah N Gangadhar BN Naga Venkatesha Murthy PJ Harish MG Subbakrishna DK Vedamurthachar A. Antidepressant efficacy of sudarshan kriya yoga (SKY) in melancholia: A randomized comparison with electroconvulsive therapy (ECT) and imipramine. J Affect Disord. (2000) 57:255–9. doi: 10.1016/S0165-0327(99)00079-8, PMID: 10708840

[33] Seppälä EM Nitschke JB Tudorascu DL Hayes A Goldstein MR Nguyen DT. Breathing-based meditation decreases posttraumatic stress disorder symptoms in U.S. military veterans: A randomized controlled longitudinal study. J Traumatic Stress. (2014) 27:397–405. doi: 10.1002/jts.21936, PMID: 25158633 PMC4309518

[34] Sureka P Govil S Dash D Dash C Kumar M Singhal V. Effect of sudarshan kriya on male prisoners with non psychotic psychiatric disorders: A randomized control trial. Asian J Psychiatry. (2014) 12:43–9. doi: 10.1016/j.ajp.2014.06.010, PMID: 25440560

[35] Vedamurthachar A Janakiramaiah N Hegde JM Shetty TK Subbakrishna DK Sureshbabu SV. Antidepressant efficacy and hormonal effects of sudarshana kriya yoga (SKY) in alcohol dependent individuals. J Affect Disord. (2006) 94:249–53. doi: 10.1016/j.jad.2006.04.025, PMID: 16740317

[36] Banushi B Brendle M Ragnhildstveit A Murphy T Moore C Egberts J. Breathwork interventions for adults with clinically diagnosed anxiety disorders: A scoping review. Brain Sci. (2023) 94(1-3):249–53. doi: 10.3390/brainsci13020256, PMID: 36831799 PMC9954474

[37] Sharpe E Lacombe A Sadowski A Phipps J Heer R Rajurkar S. Investigating components of pranayama for effects on heart rate variability. J Psychosomatic Res. (2021) 148:110569. doi: 10.1016/j.jpsychores.2021.110569, PMID: 34271528 PMC8568305

[38] Zaccaro A Piarulli A Laurino M Garbella E Menicucci D Neri B. How breath-control can change your life: A systematic review on psycho-physiological correlates of slow breathing. Front Hum Neurosci. (2018) 12:353. doi: 10.3389/fnhum.2018.00353, PMID: 30245619 PMC6137615

[39] Kox M van Eijk LT Zwaag J van den Wildenberg J Sweep FC van der Hoeven JG. Voluntary activation of the sympathetic nervous system and attenuation of the innate immune response in humans. Proc Natl Acad Sci U S A. (2014) 111:7379–84. doi: 10.1073/pnas.1322174111, PMID: 24799686 PMC4034215

[40] Haller H Ostermann T Lauche R Cramer H Dobos G. Credibility of a comparative sham control intervention for Craniosacral Therapy in patients with chronic neck pain. Complement Ther Med. (2014) 22:1053–9. doi: 10.1016/j.ctim.2014.09.007, PMID: 25453528

[41] Cramer H Lauche R Langhorst J Dobos G. Are Indian yoga trials more likely to be positive than those from other countries? A systematic review of randomized controlled trials. Contemp Clin Trials. (2015) 41:269–72. doi: 10.1016/j.cct.2015.02.005, PMID: 25705015

[42] Junqueira DR Zorzela L Golder S Loke Y Gagnier JJ Julious SA. CONSORT Harms 2022 statement, explanation, and elaboration: updated guideline for the reporting of harms in randomized trials. J Clin Epidemiol. (2023) 158:149–65. doi: 10.1016/j.jclinepi.2023.04.005, PMID: 37100738

[43] Moher D Hopewell S Schulz KF Montori V Gøtzsche PC Devereaux PJ. CONSORT 2010 Explanation and Elaboration: Updated guidelines for reporting parallel group randomised trials. J Clin Epidemiol. (2010) 63:e1–37. 20346624 10.1016/j.jclinepi.2010.03.004

[44] Ward L Nault D Cramer H Moonaz S. Development of the CLARIFY (checklist standardising the reporting of interventions for yoga) guidelines: A delphi study. Br Med J Open. (2022) 12:e054585. doi: 10.1136/bmjopen-2021-054585, PMID: 35105638 PMC8804643

[45] European Institute for Gender Equality (EIGE). Franklin P. Gender Equality Index 2021: Health. Luxembourg: Publications Office of the European Union (2021). Available online at: https://eige.europa.eu/publications/gender-equality-index-2021-health.

[46] Berenz EC Vujanovic AA Coffey SF Zvolensky MJ. Anxiety sensitivity and breath-holding duration in relation to PTSD symptom severity among trauma exposed adults. J Anxiety Disord. (2012) 26:134–9. doi: 10.1016/j.janxdis.2011.10.004, PMID: 22047652 PMC3254809

